# Clinical Factors Affecting the Rate of Liver Regeneration in Living Donors after Right Hepatectomy

**DOI:** 10.3390/jpm14050458

**Published:** 2024-04-26

**Authors:** Minkyoung Kim, Suk-Won Suh, Eun Sun Lee, Sanggyun Suh, Seung Eun Lee, Yoo Shin Choi

**Affiliations:** 1 Department of Surgery, College of Medicine, Chung-Ang University, Seoul 156-755, Republic of Korea; minkyoungkk@cauhs.or.kr (M.K.); seo861214@cauhs.or.kr (S.S.);selee508@cau.ac.kr (S.E.L.); choiys@cau.ac.kr (Y.S.C.); 2 Department of Radiology, College of Medicine, Chung-Ang University, Seoul 156-755, Republic of Korea; seraph377@cau.ac.kr

**Keywords:** living donors, remnant liver volume, operative safety, liver regeneration, intraoperative blood loss

## Abstract

Sufficient liver regeneration after a right hepatectomy is important in living donors for preventing postoperative hepatic insufficiency; however, it differs for each living donor so we investigated the clinical factors affecting the rate of liver regeneration after hepatic resection. This retrospective case–control study investigated fifty-four living donors who underwent a right hepatectomy from July 2015 to March 2023. Patients were classified into 2 groups by the remnant/total volume ratio (RTVR): Group A (RTVR < 30%, n = 9) and Group B (RTVR ≥ 30%, n = 45). The peak postoperative level of total bilirubin was more elevated in Group A than in Group B (3.0 ± 1.1 mg/dL vs. 2.3 ± 0.8 mg/dL, *p* = 0.046); however, no patients had hepatic insufficiency or major complications. The rates of residual liver volume (RLV) growth at Postoperative Week 1 (89.1 ± 26.2% vs. 53.5 ± 23.7%, *p* < 0.001) were significantly greater in Group A, and its significant predictors were RTVR (β = −0.478, *p* < 0.001, variance inflation factor (VIF) = 1.188) and intraoperative blood loss (β = 0.247, *p* = 0.038, VIF = 1.182). In conclusion, as the RLV decreases, compensatory liver regeneration after hepatic resection becomes more prominent, resulting in comparable operative outcomes. Further studies are required to investigate the relationship between hematopoiesis and the rate of liver regeneration.

## 1. Introduction

Liver transplantation (LT) has been widely performed as a curative treatment for patients with end-stage liver disease (ESLD) [1]. LT is also a preferred treatment for HCC patients because it simultaneously treats the tumor and cirrhotic liver, which is related to tumor recurrence [2]. The demand for LT is increasing; however, organs from deceased donors are limited, especially in specific regions, and living donor liver transplantation (LDLT) has become an alternative option [3]. LDLT offers a short waiting-list time and proper pre-LT treatment, which give oncologic advantages to HCC patients [4]. One of the most important factors to consider before performing LDLT is the safety of the living donors. Living donors do not benefit medically from the operation; therefore, it is ethically very important to make an effort to minimize postoperative morbidity and mortality. Since there might be potential risks for living donors, donation should only be performed after fully discussing these risks with both the donor and recipient.

Liver size is a critical factor for the selection of living donors in LDLT. Currently, the right hemiliver is mainly used as a graft to meet the demand for an adequate liver volume in adult recipients [5]. The residual liver volume (RLV) of living donors is much smaller after right liver donation, which would also be insufficient to prevent postoperative hepatic failure. With the accumulation of LDLT experience and advances in radiological techniques for measuring functional liver volume, a RTVR of at least 30% is widely accepted in current practice for donor safety [5]. In countries where LDLT was mostly performed, attempts to perform the donor hepatectomy, even if the RTVR is less than the suggested 30%, are made [6]. However, it is still controversial that living donors with a small RTVR have a risk of postoperative morbidity and mortality.

The liver is rapidly regenerated by a combination of hypertrophy and proliferation of residual liver cells following hepatic resection [7]. Sufficient liver regeneration is important to ensure good postoperative outcomes in living donors. Previous studies have shown that liver regeneration rapidly occurs in the first week after right hepatectomy in living donors. As the RLV decreases, compensation through relatively extensive regeneration becomes more prominent [8]. However, the rate of liver regeneration varies from individual to individual, even with similar types of resections considered for each living donor. Older age, male sex, high body mass index (BMI), preoperative alanine aminotransferase (ALT), and the presence of moderate or severe steatosis, have been reported to have a significant negative impact on postoperative liver regeneration in previous studies [8,9]. Therefore, it would be very important to identify the clinical factors related to liver regeneration, especially in living donors with a RTVR < 30%, to prevent postoperative liver insufficiency causing catastrophic events.

This study aimed to determine the clinical factors affecting the rate of liver regeneration in living donors after a right hepatectomy to ensure their operative safety.

## 2. Patients and Methods

### 2.1. Patients

This retrospective case–control study analyzed fifty-four living donors who underwent a right hepatectomy from July 2015 to March 2023 at our institution. The selection of living donors was made using the standardized protocol of our institution, described previously [3]. In living donors who had a RTVR < 30%, we made decisions regarding their donations using the protocol of our institution; age < 50, hepatic steatosis <10%, and no past medical or operative history of the living donors, as well as a MELD (model for end-stage liver disease) score < 38 and GRWR (graft-to-recipient weight ratio) >0.8 of the recipients (Table 1). 

The patients were classified into two groups depending on the RTVR: Group A (RTVR < 30%, n = 9) and Group B (RTVR ≥ 30%, n = 45). Clinical demographics, operative outcomes, and postoperative complications were compared between the two groups. Furthermore, RLV growth rates and liver volumetric recovery after donor hepatectomy were compared between the two groups. We also analyzed the influence of demographics and various clinical and operative variables on the rate of liver regeneration after hepatic resection.

The Institutional Review Board of our institution approved this study (IRB No. 2310-010-19492). It was performed in accordance with the Declaration of Helsinki (as revised in 2013). Informed consent was waived because it was a retrospective study in which we did not use any patient-identifying data.

### 2.2. Data Collection

The following data were collected from all patients: age, sex, history of diabetes mellitus (DM) or hypertension, BMI, and perioperative levels of total bilirubin (TB), international normalized ratio (INR), albumin, aspartate aminotransferase (AST), ALT, degree of hepatic steatosis, total liver volume (TLV), RLV, and RTVR. Operative details, such as operative duration, intraoperative blood loss, and requirement for blood transfusion, were collected. Postoperative liver insufficiency was diagnosed if the peak postoperative TB level was more than 7 mg/dL and/or the ascites present totaled more than 500 mL per day [10]. Postoperative complications were classified using a modified version of the Clavien system, which has been previously described [11]. The incidence of rehospitalization and the length of intensive care unit admission and postoperative hospital stay were investigated. 

**Figure 1:**
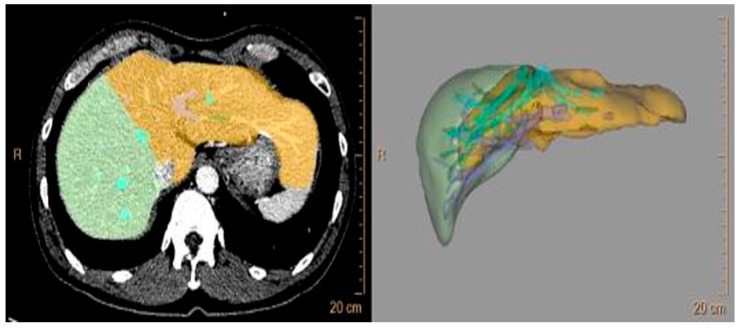
Liver volumetry. Three-dimensional reconstruction of the liver was rendered with preoperative liver dynamic computed tomography (CT) using a software program (TeraRecon Acuaris iNtuition version 4.4.12).

Three-dimensional reconstruction of the right and left liver was performed with preoperative liver dynamic computed tomography (CT) using a software program (TeraRecon Acuaris iNtuition version 4.4.12) to estimate the graft volume (GV) and RTVR (Figure 1). Major vessels, including the inferior vena cava, first-order branches of the portal and hepatic veins, and major fissures were excluded by tracing. To minimize errors, each volume measurement was performed twice, and the average value was calculated. We measured the liver regeneration volume at Postoperative Week 1 (POW 1) and Postoperative Month 3 (POM 3) using the same method. The RLV growth rates were calculated as the ratio of the estimated liver volume to the preoperative RLV. Liver volumetric recovery was defined as follows:

Liver volumetric recovery (%) = estimated liver volume/preoperative TLV × 100

### 2.3. Anesthetic and Surgical Techniques

The standardized anesthetic and surgical techniques were described in a previous report [12]. General anesthesia was induced with intravenous fentanyl, propofol, and to start endotracheal tube insertion. It was maintained with sevoflurane, nitrous oxide, and oxygen with an intravenous Rocuronium, a muscle relaxant. After intubation, patients had central catheterization guided by ultrasonography. No preoperative fluid was infused and it was also minimized after the start of surgery, maintaining a CVP of less than 5 mmHg. After completion of hepatic parenchymal transection, the crystalloid fluid (10 to 12 mL/kg/h) was infused to replace the perioperative fluid deficit. A colloid solution was also administrated to preserve volume status. We administered a vasopressor drug (mostly 5 mg bolus of ephedrine) if the mean arterial pressure decreased below 60 mmHg. And phenylephrine (50 mcg bolus) was used when the heart rate was elevated. Red blood cells were transfused if the level of hemoglobin concentration was lower than 7 g/dL in the postoperative period. Decisions regarding ICU admission were made by considering the condition of patients that required inotropic agents. 

All the hepatic resections were performed by the same method. Parenchymal transection was performed using an ultrasound aspirator with preservation of the middle hepatic vein in the donor side of the liver. The middle hepatic veins (>5 mm in length) were typically reconstructed using an artificial graft.

### 2.4. Statistical Analysis

The correlation between the estimated and actual GV was evaluated using Pearson’s coefficient. Student’s *t*-test or the Kruskal–Wallis test was performed for the analysis of normally distributed data. χ^2^ test, Fisher’s exact test, or general linear model analysis of variance were used for comparisons of descriptive data. Multivariate analysis with a linear regression model was performed to investigate the predictive factors for liver regeneration at POW 1. To rule out multicollinearity issues, the variance inflation factor (VIF) was used. The VIF measures the degree of correlation between a variable and the remaining variables in the model. If a *p*-value < 0.05, it had a statistical significance. SPSS version 19.0 was used for the analysis (IBM Corp., Armonk, NY, USA).

## 3. Results

### 3.1. Correlation between the Estimated GV and Actual Graft Weight

**Figure 2:**
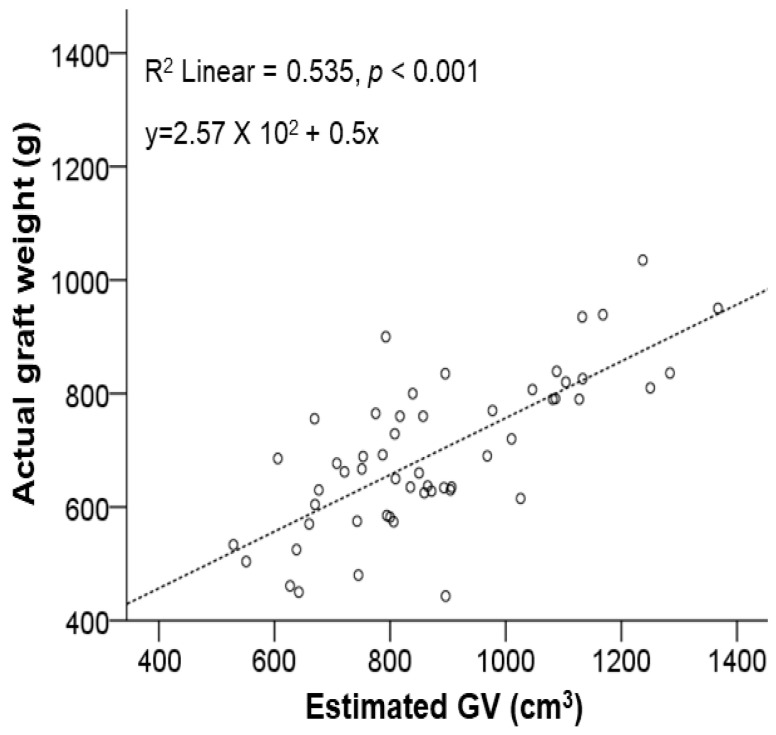
Scatter plots showing correlations between the estimated graft volume (GV) and actual graft weight. They showed a significant correlation (R^2^ = 0.535, *p* < 0.001).

A comparison of the estimated GV by volumetric assessment using preoperative CT and the actual graft weight intraoperatively measured after graft extraction showed a significant correlation (R^2^ = 0.535, *p* < 0.001; Figure 2). 

### 3.2. Demographics

The preoperative TB level was significantly higher in Group B (0.4 ± 0.2 mg/dL vs. 0.6 ± 0.2 mg/dL, *p* = 0.030). The mean TLV was smaller in Group A, compared to Group B; however, it was not statistically significant (1270 ± 289 cm^3^ vs. 1391 ± 300 cm^3^, *p* = 0.274). The mean RLV (379 ± 95 cm^3^ vs. 515 ± 116 cm^3^, *p* = 0.002) was smaller and the media RTVR (29.6% (range 27.8 to 29.9%) vs. 36.7% (range 31.9 to 46.1%), *p* < 0.001) was lower in Group A than in Group B, with statistical significance (Table 2). 

### 3.3. Operative Outcomes

The mean operative duration showed no significant difference between the two groups (335 ± 62 min vs. 310 ± 46 min, *p* = 0.665). Group A had a higher intraoperative blood loss than that of Group B, without statistical significance (467 ± 255 mL vs. 343 ± 160 mL, *p* = 0.062). None of the living donors in either group underwent intraoperative blood transfusions. There were no significant differences in postoperative laboratory results, such as the peak levels of INR, AST, and ALT, and the lowest level of albumin; however, the peak TB level showed a significant difference (3.0 ± 1.1 mg/dL vs. 2.3 ± 0.8 mg/dL, *p* = 0.046). None of the patients in either group developed postoperative hepatic insufficiency and did not require intensive care unit admission. No significant differences were found in the postoperative hospital stay (10.2 ± 1.9 days vs. 10.7 ± 3.4 days, *p* = 0.158) and re-hospitalization rate (0 vs. 1 (2.2%), *p* = 0.652) between the two groups (Table 3). 

### 3.4. Postoperative Complications

No significant differences in postoperative complications were found between the two groups (26.4% vs. 31.1%, *p* = 0.896). All of the complications were minor and Grade 1, including transient bile leakage, pleural effusion, and paralytic ileus, and Grade 2, such as wound infection and prolonged ascites (>7 days). However, there were also no significant differences in incidences between the two groups. There was no major postoperative complication (≥grade 3) observed in the two groups (Table 4).

### 3.5. Rate of RLV Growth and Liver Volumetric Recovery following Donor Hepatectomy

**Figure 3:**
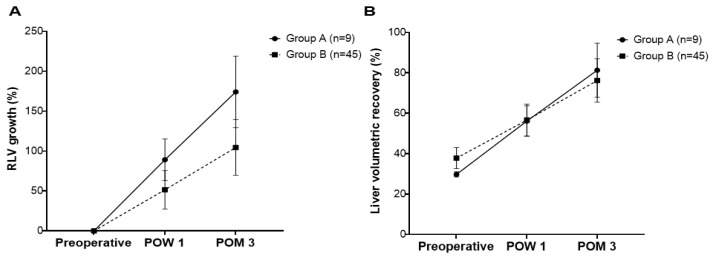
The rates of residual liver volume (RLV) growth (**A**) and liver volumetric recovery (**B**). The rates of RLV growth at Postoperative Week 1 (POW 1; 89.1 ± 26.2% vs. 53.5 ± 23.7%, *p* < 0.001) and Postoperative Month 3 (POM 3; 174.1 ± 44.7% vs. 104.6 ± 34.7%, *p* < 0.001) were significantly greater in Group (**A**) than in Group (**B**). The liver volumetric recovery was similar between the two groups at POW 1 (56.1 ± 7.5% vs. 56.3 ± 7.8%, *p* = 0.925) and greater in Group (**A**) than in Group (**B**) at POM 3 (81.3 ± 13.4% vs. 76.2 ± 10.7%, *p* = 0.220); however, the differences were not statistically significant.

The rates of RLV growth at POW 1 (89.1 ± 26.2% vs. 53.5 ± 23.7%, *p* < 0.001) and POM 3 (174.1 ± 44.7% vs. 104.6 ± 34.7%, *p* < 0.001) were significantly greater in Group A than in Group B (Figure 3A). The liver volumetric recovery was similar between the two groups at POW 1 (56.1 ± 7.5% vs. 56.3 ± 7.8%, *p* = 0.925) and greater in Group A at POM 3 than in Group B (81.3 ± 13.4% vs. 76.2 ± 10.7%, *p* = 0.220), but the differences were not statistically significant (Figure 3B). 

### 3.6. Factors Predictive of Liver Regeneration at POW 1

**Figure 4:**
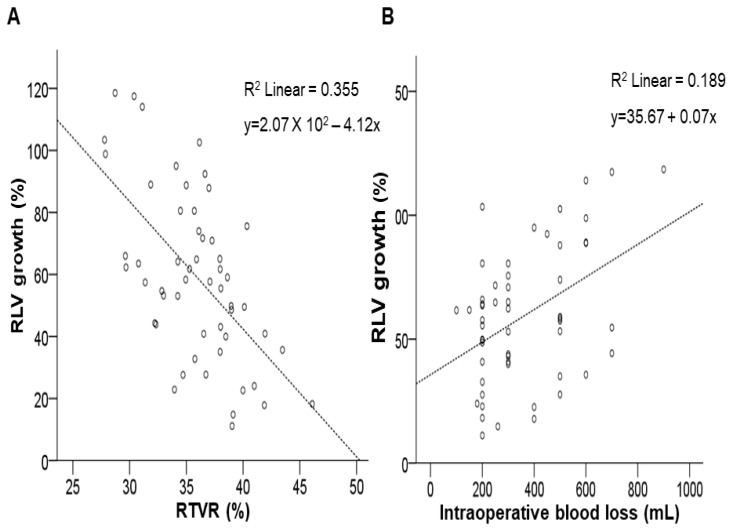
Scatter diagram showing the correlation of significant variables, such as the remnant/total volume ratio (RTVR) (**A**) and intraoperative blood loss (**B**), with regeneration of the liver at Postoperative Week 1 (POW 1).

We identified factors predictive of liver regeneration at POW 1. Preoperative TB (*β* = −0.303, *p* = 0.026), RTVR (*β* = −0.599, *p* < 0.001), intraoperative blood loss (*β* = 0.435, *p* = 0.001), and postoperative peak AST (*β* = 0.337, *p* = 0.013) were significantly related with liver regeneration at POW 1 following donor hepatectomy in the univariate analysis. The only significant factors predictive of liver regeneration at POW 1 in the multivariate analysis were RTVR (*β* = −0.478, *p* < 0.001, VIF = 1.188) and intraoperative blood loss (*β* = 0.247, *p* = 0.038, VIF = 1.182; Table 5). The scatter plot correlation of significant factors, such as RTVR (R^2^ = 0.355, *p* < 0.001) and intraoperative blood loss (R^2^ = 0.189, *p* = 0.038) with the rate of RLV growth at POW 1, is shown in Figure 4.

## 4. Discussion

Living donors with a RTVR < 30% had comparable operative outcomes in this study. In addition, RTVR and intraoperative blood loss were identified as significant factors affecting the rate of liver regeneration after hepatic resection in living donors. 

Donor safety after living donor hepatectomy is a major concern in the planning of LDLT. To ensure donor safety after liver surgery, it is necessary to secure a certain RLV. It is well known that leaving a RLV of 30% or more can ensure operative safety in the current practice [5]. One study reported that the operation of living donors with a RTVR < 30% could be safely performed if we carefully selected the candidates [6]. In this study, we also used similar criteria considering age, hepatic steatosis, and medical and operatory history of living donors and the MELD score and proper graft size to recipients to select living donors with a RTVR < 30%, and they showed comparable operative outcomes. However, one had reported the adverse effects on postoperative outcomes in donors with small RLVs [13]. Along with the RLV, the low remnant liver volume-to-donor body weight ratio (RLVBWR) was also suggested to be a significant predictor of the metabolic demand for liver regeneration [8].

Several studies have been conducted to identify factors that may influence liver regeneration after donor hepatectomy [8,9,13]. The incidence of postoperative major complications after liver donation in older donors was significantly higher than that of younger donors [3]. However, nowadays, we are faced with an older and healthier population than before, and many reports have shown comparable operative outcomes between older and younger donors [14]. The upper age limit for living donors at our institution is 65 years and age did not affect the rate of liver regeneration or postoperative outcomes in this study. The preoperative ALT level, related to fatty liver disease was reported to be a significant predictor for liver regeneration. Hepatic steatosis caused marked impairments in regeneration and an inability to tolerate ischemic injury [8]. In our institution, donors with hepatic steatosis, more than 10% were recommended to reduce their weight with a protein-rich diet and exercise for 4 to 6 weeks. Most of these donors had an improvement in hepatic steatosis and underwent donor operations without any significant complications. None of the living donors had hepatic steatosis > 10% at the time of donor hepatectomy in this study. The RLV is thought to affect liver regeneration after resection; a small RTVR in donors leads to an increased release of cytokines and growth factors, which promote regeneration of the remnant liver [15]. This study also showed that a small RLV was identified as a predictor of liver regeneration at POW 1. One study reported that high portal venous velocity is an important hemodynamic factor for the rate of liver regeneration after right donor hepatectomy. It might be related to cytokines inducing hepatocytes to enter the cell cycle, such as tumor necrosis factor (TNF) and interleukin (IL)-6 [9]. We did not investigate this relationship, but a high portal venous velocity may also be related to a small RLV in the early postoperative period. 

In this study, intraoperative blood loss was a significant factor influencing liver regeneration after hepatectomy. One possible explanation is that bleeding during donor hepatectomy stimulates erythropoiesis, which promotes liver regeneration. Erythropoietin stimulates erythropoiesis and is secreted in response to chronic anemia and acute hemorrhage, including intraoperative blood loss, promoting the differentiation of erythroid progenitor cells. Increased liver regeneration by stimulation of erythropoiesis is related to increments of Ki-67 proliferation, hepatocyte growth factor (HGF), IL-6, vascular endothelial growth factor (VEGF), and IL-6 [16]. Perioperative erythropoietin has been suggested to stimulate liver regeneration after hepatectomy [17]. Therefore, this should be considered in living donors with small RLVs to promote postoperative liver regeneration. Other liver sections including the left lobe with the caudate lobe, the right posterior segment, the left tri-segment, or the dual grafts could be considered to be used as a liver graft. to increase both donor safety and graft volume to meet the needs of recipients. 

This study has some limitations. First, a comparison of the recipient outcomes between the two groups would increase the impact of this study. However, it was a retrospective study, completely dependent on the medical records of our institution. Second, the study population was disproportionate between the groups. Propensity score matching would be an alternative method for improving the accuracy of the statistical differences, but the study population was relatively small, and thus, this method was also unsuitable. Thus, in the future, prospective studies with a large population are required to clarify the operative safety of living donors with a small remnant liver. Finally, in future studies, liver regeneration due to differences in the RTVR and intraoperative blood loss may be quantified by analyzing changes in related cytokines, such as HGF, VEGF, and IL-6.

## 5. Conclusions

Selective living donors with a RTVR < 30% had comparable operative outcomes with compensative liver regeneration. RTVR and intraoperative blood loss are significant factors affecting the rate of liver regeneration after hepatic resection in living donors. These clinical factors would be very important especially in living donors with a RTVR < 30% to predict the rate of liver regeneration after donor hepatectomy and prevent postoperative morbidity and mortality. Further studies are required to ensure operative safety in living donors with a small RLV. 

## Figures and Tables

**Table 1:** Selection criteria of living donors with a RTLV < 30%.

Living Donor	Recipient
Age < 50	MELD < 38
Hepatic steatosis < 10%	GRWR > 0.8
No past medical or operative history	

MELD, model for end-stage liver disease; GRWR, graft-to-recipient weight ratio.

**Table 2:** Demographics of living donors.

	Group A (n = 9) (RTVR < 30%)	Group B (n = 45) (RTVR ≥ 30%)	*p*
Age (years)	37.5 (22–46)	38.7 (22–65)	0.559
Sex (male)	5 (55.6%)	26 (57.8%)	0.902
BMI (kg/m^2^)	24.2 (±2.1)	23.7 (±3.1)	0.688
DM	0	3 (6.7%)	0.425
HTN	0	2 (4.4%)	0.519
Preoperative TB (mg/dL)	0.4 (±0.2)	0.6 (±0.2)	0.030
Preoperative INR	1.03 (±0.06)	1.04 (±0.06)	0.690
Preoperative albumin (mg/dL)	4.4 (±0.5)	4.5 (±0.3)	0.354
Preoperative AST (IU/L)	19 (±3)	23 (±10)	0.350
Preoperative ALT (IU/L)	14 (±2)	21 (±14)	0.154
Hepatic steatosis (%)	2 (0–9)	2 (0–10)	0.960
TLV (cm^3^)	1270 (±289)	1391 (±300)	0.274
RLV (cm^3^)	379 (±95)	515 (±116)	0.002
RTVR (%)	29.6 (27.8–29.9)	36.7 (31.9–46.1)	<0.001

DM, diabetes mellitus; HTN, hypertension; TB, total bilirubin; INR, international normalized ratio; AST, aspartate aminotransferase; ALT, alanine aminotransferase; TLV, total liver volume; RLV, residual liver volume; RTVR, remnant/total volume ratio.

**Table 3:** Operative outcomes of living donors.

	Group A (n = 9) (RTVR < 30%)	Group B (n = 45) (RTVR ≥ 30%)	*p*
Operative duration (min)	335 (±62)	310 (±46)	0.665
Intraoperative blood loss (mL)	467 (±255)	343 (±160)	0.062
Blood transfusion	0	0	NS
Postoperative laboratory results			
Peak TB (mg/dL)	3.0 (±1.1)	2.3 (±0.8)	0.046
Peak INR	1.57 (±0.14)	1.47 (±0.15)	0.065
Lowest albumin (mg/dL)	2.9 (±0.2)	3.1 (±0.3)	0.144
Peak AST (IU/L)	173 (±80)	171 (±53)	0.920
Peak ALT (IU/L)	162 (±98)	168 (±56)	0.807
Postoperative hepatic insufficiency	0	0	NS
Intensive care unit admission (%)	0	0	NS
Postoperative hospital stay (days)	10.2 (±1.9)	10.7 (±3.4)	0.158
Rehospitalization (%)	0	1 (2.2%)	0.652

TB, total bilirubin; INR, international normalized ratio; AST, aspartate aminotransferase; ALT, alanine aminotransferase; RTVR, remnant/total volume ratio.

**Table 4:** Postoperative complications of living donors.

	Group A (n = 9) (RTVR < 30%)	Group B (n = 45) (RTVR ≥ 30%)	*p*
Postoperative complications (%)	3 (26.4%)	14 (31.1%)	0.896
Grade 1			
Transient bile leakage	0	1 (2.2%)	0.652
Pleural effusion	1 (11.1%)	3 (7.3%)	0.704
Paralytic ileus	1 (11.1%)	6 (13.3%)	0.856
Grade 2			
Wound infection	1 (11.1%)	4 (8.9%)	0.652
Prolonged ascites (>7 days)	1 (11.1%)	1 (2.2%)	0.197
Grade 3	0	0	NS
Grade 4	0	0	NS
Grade 5	0	0	NS

RTVR, remnant/total volume ratio.

**Table 5:** Univariate and multivariate analysis of factors predictive for liver regeneration at POW 1.

	Univariate Analysis		Multivariate Analysis		
Variable	*β*	*p*	*β*	*p*	VIF
Age (year)	0.057	0.680			
Sex (male)	0.082	0.556			
BMI (kg/m^2^)	0.054	0.696			
Diabetes mellitus	−0.218	0.114			
Hypertension	0.003	0.980			
Preoperative TB (mg/dL)	−0.303	0.026			
Preoperative INR	−0.146	0.293			
Preoperative albumin (mg/dL)	0.090	0.519			
Preoperative AST (IU/L)	−0.103	0.456			
Preoperative ALT (IU/L)	−0.126	0.364			
Hepatic steatosis (%)	−0.196	0.154			
RTVR (%)	−0.599	<0.001	−0.478	<0.001	1.188
Operation time (min)	0.197	0.154			
Intraoperative blood loss (mL)	0.435	0.001	0.247	0.038	1.182
Postoperative peak TB (mg/dL)	−0.070	0.616			
Postoperative peak INR	−0.120	0.387			
Postoperative lowest albumin (mg/dL)	0.044	0.753			
Postoperative peak AST (IU/L)	0.337	0.013			
Postoperative peak ALT (IU/L)	0.180	0.193			
Postoperative complications	−0.129	0.352			

POW 1, Postoperative Week 1; TB, total bilirubin; INR, international normalized ratio; AST, aspartate aminotransferase; ALT, alanine aminotransferase; RTVR, remnant/total volume ratio; VIF, variance inflation factor.

## Data Availability

Data are available from the authors upon request.

## Abbreviations

ALT	alanine aminotransferase
AST	aspartate aminotransferase
BMI	body mass index
CT	computed tomography
DM	diabetes mellitus
ESLD	end-stage liver disease
GV	graft volume
INR	international normalized ratio
LDLT	living donor liver transplantation
LT	liver transplantation
POM	postoperative month
POW	postoperative week
RLV	residual liver volume
RTVR	remnant/total volume ratio
TB	total bilirubin
TLV	total liver volume
VIF	variance inflation factor
